# Genetic diversity and seroprevalence of *Toxoplasma gondii* in COVID‑19 patients; a first case-control study in Iran

**DOI:** 10.1186/s12879-023-08964-9

**Published:** 2024-01-03

**Authors:** Mehdi Hasanzadeh, Ehsan Ahmadpour, Mahmoud Mahami-Oskouei, Saeed Musavi, Mahdi Parsaei, Nazila Sarafraz, Adel Spotin

**Affiliations:** 1 Immunology Research Center, Tabriz University of Medical Sciences, Tabriz, Iran; 2 Department of Parasitology and Mycology, School of Medicine, Tabriz University of Medical Sciences, Tabriz, Iran; 3 Infectious and Tropical Disease Research Center, Tabriz University of Medical Sciences, Tabriz, Iran; 4 Department of Statistics and Epidemiology, Faculty of Health, Tabriz University of medical science, Tabriz, Iran; 5 Vice chancellor for health, Tabriz University of Medical Sciences, Tabriz, Iran

**Keywords:** Toxoplasmosis, COVID‑19 patients, Sequencing, Serology, Northwest Iran

## Abstract

**Background:**

Toxoplasmosis is a serious or life-threatening disease in immunosuppressed patients and pregnant women. This study examined the likely association between *Toxoplasma gondii* infection and COVID-19 patients with moderate illness.

**Methods:**

Seventy blood samples were collected from patients at the Health Reference Laboratory of Tabriz, Northwest Iran from April 2021 to September 2021. In addition, 70 healthy subjects of the same age (37 ± 15 years) and sex distribution were ethnically matched. Sera samples were examined for the detection of anti*-Toxoplasma* antibodies using ELISA. Nested-PCR targets were amplified based on the B1 and GRA6 genes. GRA6 amplicons were subjected to sequencing and phylogenetic analysis.

**Results:**

The seroprevalence of toxoplasmosis based on IgG titer was 35.7% in the COVID‑19 patients and 27.1% in the control group, representing not to be associated with the *Toxoplasma* seropositivity in COVID‑19 patients (*P* = 0.18) compared to healthy subjects. Anti-*T. gondii* IgM was not found in any of the patients and healthy individuals. According to PCR amplification of the B1 and GRA6 genes, the frequency of *T. gondii* in COVID-19 patients was 14.2% (10/70). However, no *T. gondii* infection was detected in the healthy group. The CD4^+^T cell count was relatively lower in toxoplasmosis-infected patients (430–450 cells/mm3) than in control group (500–1500 cells/mm3). High genetic diversity (Hd: 0.710) of the type I strain of *T. gondii* was characterized in the patients. Present results showed that consumption of raw vegetables and close contact with stray cats can increase the transmission of *T. gondii* to COVID-19 patients (*P* < 0.01).

**Conclusions:**

The current study revealed that *T. gondii* type I infection is unequivocally circulating among the COVID-19 patients in Tabriz; However, no significant association was observed between the occurrence of *Toxoplasma* and the severity of COVID-19. To make more accurate health decisions, multicenter investigations with a larger sample size of different ethnic groups of the Iranian population are needed.

**Supplementary Information:**

The online version contains supplementary material available at 10.1186/s12879-023-08964-9.

## Introduction

*Toxoplasma gondii* as an intracellular parasite can infect approximately one-third of the residents worldwide [1]. Nearly, 39.3% of the population of Iran and 35.1-41% of people in the Northwest Iran are infected with toxoplasmosis [2, 3]. Latent toxoplasmosis has long been considered asymptomatic in immunocompetent people, but it can result in serious or life-threatening disease in patients with haematologic malignancy, immunocompromised cancer patients, organ transplants and pregnant women [1, 4–6]. Toxoplasmosis is also associated with various autoimmune [7] and inflammatory diseases [8] as well as some cancers [9, 10].

The first cases of coronavirus disease 19 (COVID-19) with an unknown source were reported in Wuhan, China [11]. SARS-CoV-2 has been shown to impair immune responses and uncontrolled inflammatory responses “cytokine storm” in severe and critical patients with COVID-19. Based on the clinical presentation (from asymptomatic to severe) of mixed infections of COVID-19 with other pathogens, including influenza A [12], *Streptococcus pneumoniae* [13] fungal [14] and *T. gondii* [15], indicating these infections could increase the severity of COVID-19. The co-occurrence of COVID-19 and toxoplasmosis as emerging intracellular infections in immunosuppressed individuals may lead to a higher incidence of mental and physical health problems [15–17].

Conflicting results regarding the association between *T. gondii* and COVID-19 have been discussed by several researchers. Some evidence also suggests that COVID-19 can reactivate latent *T. gondii* [18, 19]. However, other researchers claim that latent *T. gondii* infection can trigger suppressor effects and reduce the severity of COVID-19 through overexpression of interferons (I and II) [20, 21]. Therefore, due to the potential impact of toxoplasmosis on COVID-19 mortality, further studies are needed to clarify these apparent contradictions.

In terms of clinical significance, *T. gondii* infection can progress from the latent phase to the active phase through a decrease in CD8^+^ and CD4^+^ cells, which may lead to dangerous complications in COVID-19 patients [15, 22]. The possible association between latent toxoplasmosis and COVID-19 patients has been characterized worldwide [16, 20, 21, 23]. However, there is no case-control study on the genetic diversity of *Toxoplasma* co-infection in Iranian COVID-19 patients. The current investigation aimed to examine the probable association between toxoplasmosis and COVID-19 using serological and molecular methods in northwest Iran.

## Materials and methods

### Ethical approval, inclusion and exclusion criteria

All COVID-19 patients signed an informed consent and completed a questionnaire that included demographic data and their behavioral characteristics. This study was approved by the ethical committee of Tabriz University of Medical Sciences and followed the Helsinki’s Declaration (approval number: IR.TBZMED.REC.1400.537). In this study, patients with respiratory distress whose COVID-19 test was negative were excluded from the study. A positive COVID-19 PCR was the inclusion criteria in the current study. The control group consisted of people who had not been infected with the COVID-19 virus or had any immunodeficiency diseases.

### Study area, participants and blood sampling

**Fig. 1:**
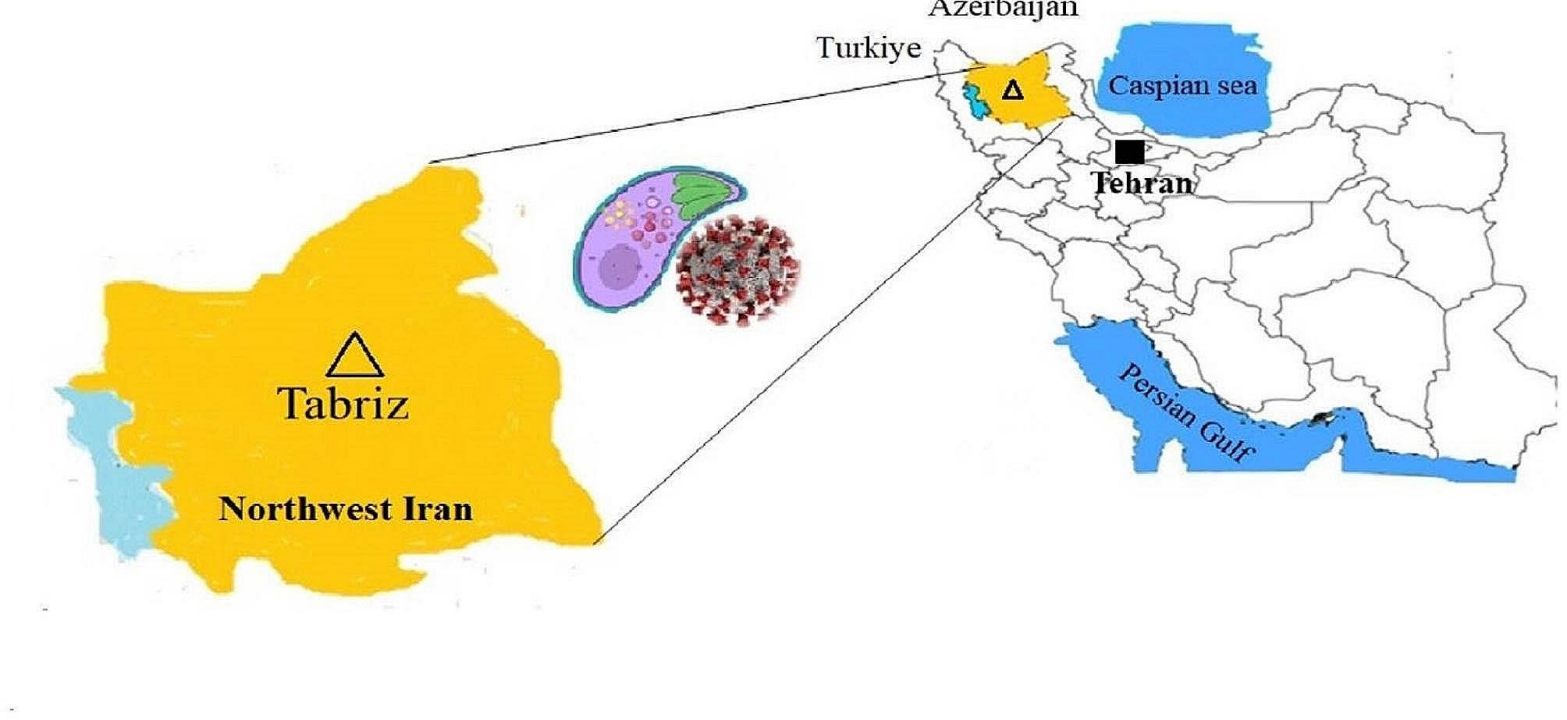
Map of Iran representing the study location (Tabriz, Northwest Iran)

Seventy blood samples were collected from patients with a real-time PCR-positive test for SARS-CoV-2 referred to Health Reference Laboratory of Tabriz, Northwest Iran from April 2021 to September 2021 (Fig. 1). As well, 70 healthy subjects of the same age and sex distribution were ethnically matched. Five ml blood samples were collected from each case and control groups into k2-EDTA (dipotassium ethylenediaminetetraacetic acid as anticoagulant) vials and collected plasma was separated into sterile plain tubes. Then, buffy coat layer and plasma were extracted and stored at -20 °C and transferred to the Health Reference Laboratory for further examination. Omicron was the predominant strain of COVID-19 in the region. None of the patients and healthy people had received the COVID-19 vaccination, corticosteroid therapy or anti-coronavirus drugs in the past.

### Serological test

Sera samples obtained from all participants (case and control groups) were detected for both anti-*T. gondii* IgG and IgM antibodies using an ELISA kit (Pishtazteb Co). IgG titers > 1.1 were considered positive.

### Nested-PCR targeting T. gondii B1 and GRA6 genes

The genomic DNA of *T. gondii* was extracted from 140 buffy coat samples (DNA extraction Kit, Yekta Tajhiz Azma, Iran). The B1 gene of *T. gondii* was amplified by nested-PCR. The fragments of 287 bp and 194 bp were amplified using a pair of external and internal primers, respectively [24]. The PCR thermal cycling conditions reported previously [25]. In addition, *T. gondii* GRA6 gene (product size: 791 bp) was identified by PCR method. PCR primer sequences and thermal cycling conditions for GRA6 gene were previously described [26].

### Sequencing and phylogenetic analysis

PCR products of *T. gondii* GRA6 gene (n: 6) were successfully sequenced (Codon Genetic Group, Iran) to detect the genotype of *T. gondii*. The chromatograms were trimmed and edited based on RefSeq (Accession number: **KX78158**) by the Sequencher 5.4.6. To display codon substitutions, a multiple sequence alignment (MSA) was drawn by BioEdit software. To validate taxonomic position of *T. gondii* genotypes, a phylogenetic tree based on the Maximum Likelihood algorithm was constructed using MEGA 5.0 software. The topology of the phylogenetic tree was validated by bootstrap values higher than 60%. *Hammondia hammondi* was addressed as an out-group index. Haplotype (genetic) diversity (Hd) and nucleotide diversity (Nd; π) were estimated using DnaSP software [27]. In this study, GRA6 sequences for the *T. gondii* isolates were deposited in the GenBank database under Accession nos; **OR193704**–**OR193706**.

### Statistical analysis

Fisher’s exact test was used to compare frequencies between groups and evaluate the correlation between characteristics of toxoplasmosis in COVID-19 patients. A multiple multinomial logistic regression model was used to assess the correlation between COVID-19 severity and anti*-T. gondii* IgG findings.

## Results

### Sociodemographic, clinical findings and risk factors

In the present study, 70 PCR-positive COVID-19 patients with moderate illness and 70 healthy individuals were examined in the Health Reference Laboratory of Tabriz. Among the patients, 80% were female and 20% were male. The mean age of patients was 36 ± 15 years. In the healthy group, 78.5% were female and 21.5% were male. The mean age of healthy group was 37 ± 15 years.

Signs and symptoms of those suffering from COVID-19 included fever > 38 °C, cough, shortness of breath, body pain, diarrhea, headache and moderate respiratory distress (means oxygen saturation in the resting state ≥ 93%). Clinical manifestations due to acquired toxoplasmosis (chorioretinitis and lymphadenopathy) were also not observed in COVID-19 patients. In addition, there was no history of death in toxoplasmosis-infected Covid-19 patients. No significant association was found between *Toxoplasma* IgG results with COVID-19 severity.

Logistic analysis showed that *T. gondii* was associated with the consumption of raw vegetables and close contact with stray cats in COVID-19 patients (*P* = 0.001–0.008) (Table 1). However, the incidence of *T. gondii* infection in COVID-19 patients was not associated with age, gender, education and consumption of raw/undercooked meat (*P* > 0.05).

**Table 1:** Behavioral characteristics and *Toxoplasma gondii* infection in COVID-19 patients and control group

Characteristic	Prevalence of *T. gondii* infection in COVID-19 patients		Prevalence of *T. gondii* infection in control group		COVID-19 patients vs. Control group		
	No. of tested	IgG Positive No (%)	No. of tested	IgG Positive No (%)	P	Confidence interval (CI)	OR
**Close contact with stray cats**	70	19(86.3)	70	2(128.5)	0.001	0.006–0.082	0.022
**Consumption of raw vegetable**	70	23(50)	70	10(27)	0.008	0.000-0.001	0.001
**Prevalence of IgG**	70	25(35 0.7)	70	19(27.1)	0.18	0.00–1.00	0.01

### Serological findings

The seroprevalence of toxoplasmosis based on IgG titer was 35.7% (25/70) in the COVID‑19 patients and 27.1% (19/70) in the control group, representing not to be associated with the *Toxoplasma* seropositivity in COVID‑19 patients (*P* = 0.18, CI: 0.00–1.00, OR: 0.01) compared to healthy subjects (Table 1). Anti-*T. gondii* IgM was not found in any of the patients and healthy individuals.

### Phylogenetic analysis and alignment

**Fig. 2:**
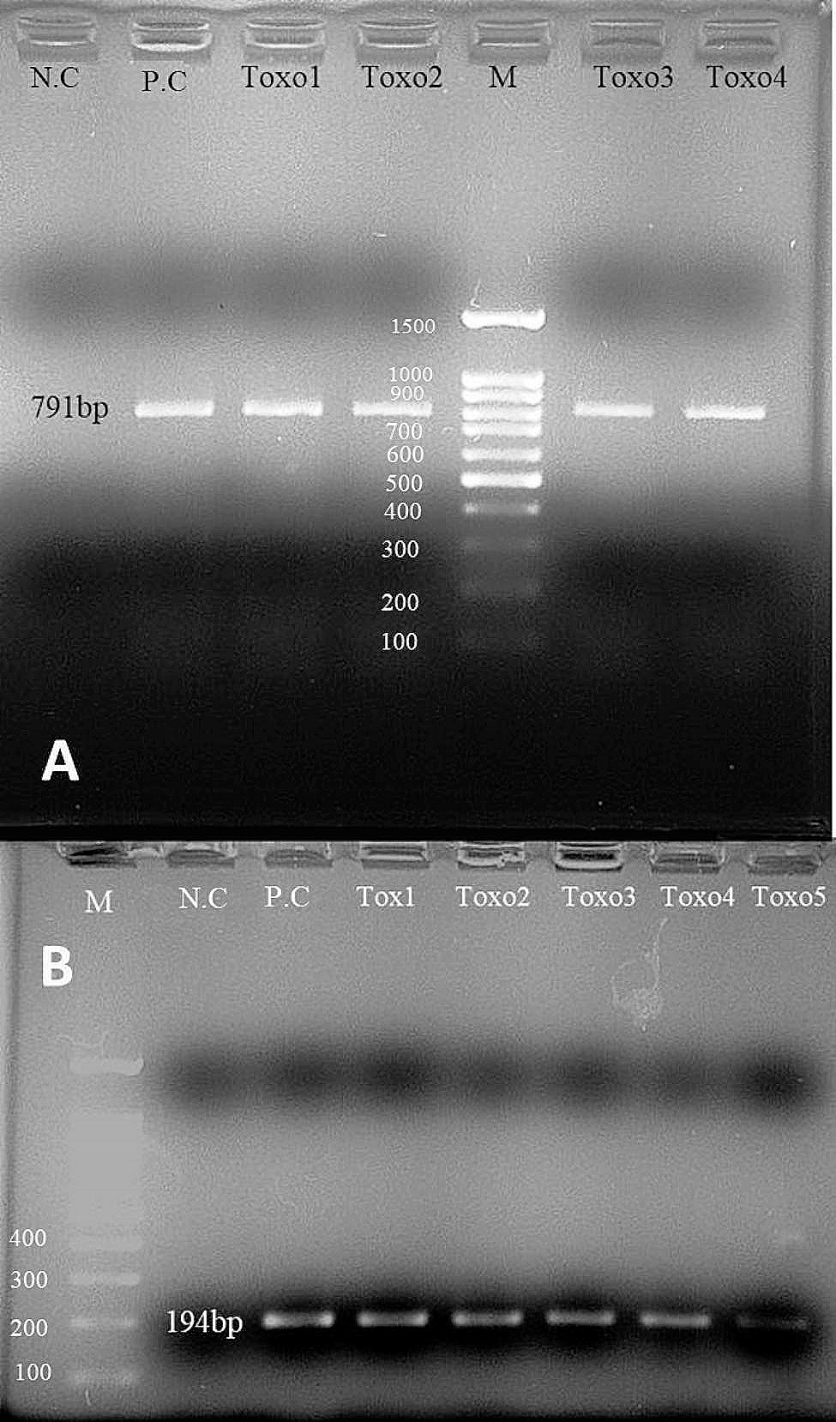
Single round-PCR assay targeting *T. gondii* GRA6 (**A**: Toxo1-Toxo4: 791 bp) and nested-PCR assay targeting *T. gondii* B1 (**B**: Toxo1-Toxo5: 194 bp). M; Ladder marker (size marker: 100 bp), P.C; positive control, N.C; negative control

In this study, ten *T. gondii* isolates were successfully amplified using the GRA6 and B1 genes in COVID-19 patients, in which no sera samples were positive for both anti-*Toxoplasma* IgG and IgM antibodies and three sera samples had a high titer of positive IgG. In this study, flow cytometry results showed that the CD4^+^T cell count in toxoplasmosis-infected COVID-19 patients was partially lower (430–450 cells/mm^3^) than in healthy subjects (500–1500 cells/mm^3^). According to PCR amplification of the B1 (194 bp) and GRA6 (791 bp) genes (Fig. 2), the frequency of *T. gondii* in COVID-19 patients was 14.2% (10/70). However, *T. gondii* infection in the healthy group was not detected by PCR.

**Fig. 3:**
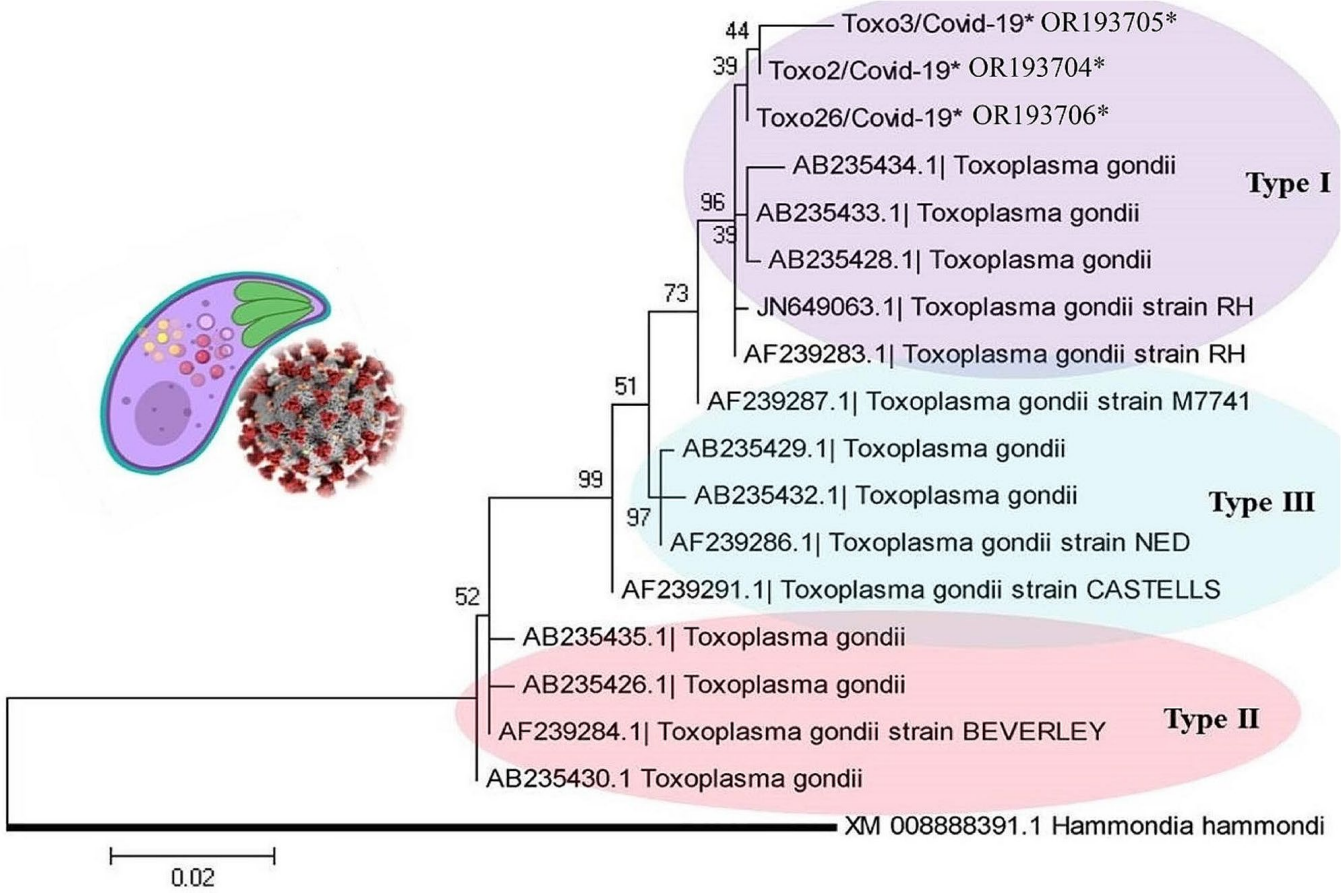
Phylogenetic tree drawn by various types (I-III) of *T. gondii* using the GRA6 gene based on the Maximum Likelihood algorithm with a Kimura 2-parameter model. The distance scale was estimated 0.02. *T. gondii* (Toxo2/3/26-COVID-19*; Accession numbers; **OR193704**–OR193706*) obtained from COVID-19 patients marked by an asterisk (*). *Hammondia hammondi* was considered as an out-group branch. Bootstrap values of higher than 60% were supported the topology on each branch

**Fig. 4:**
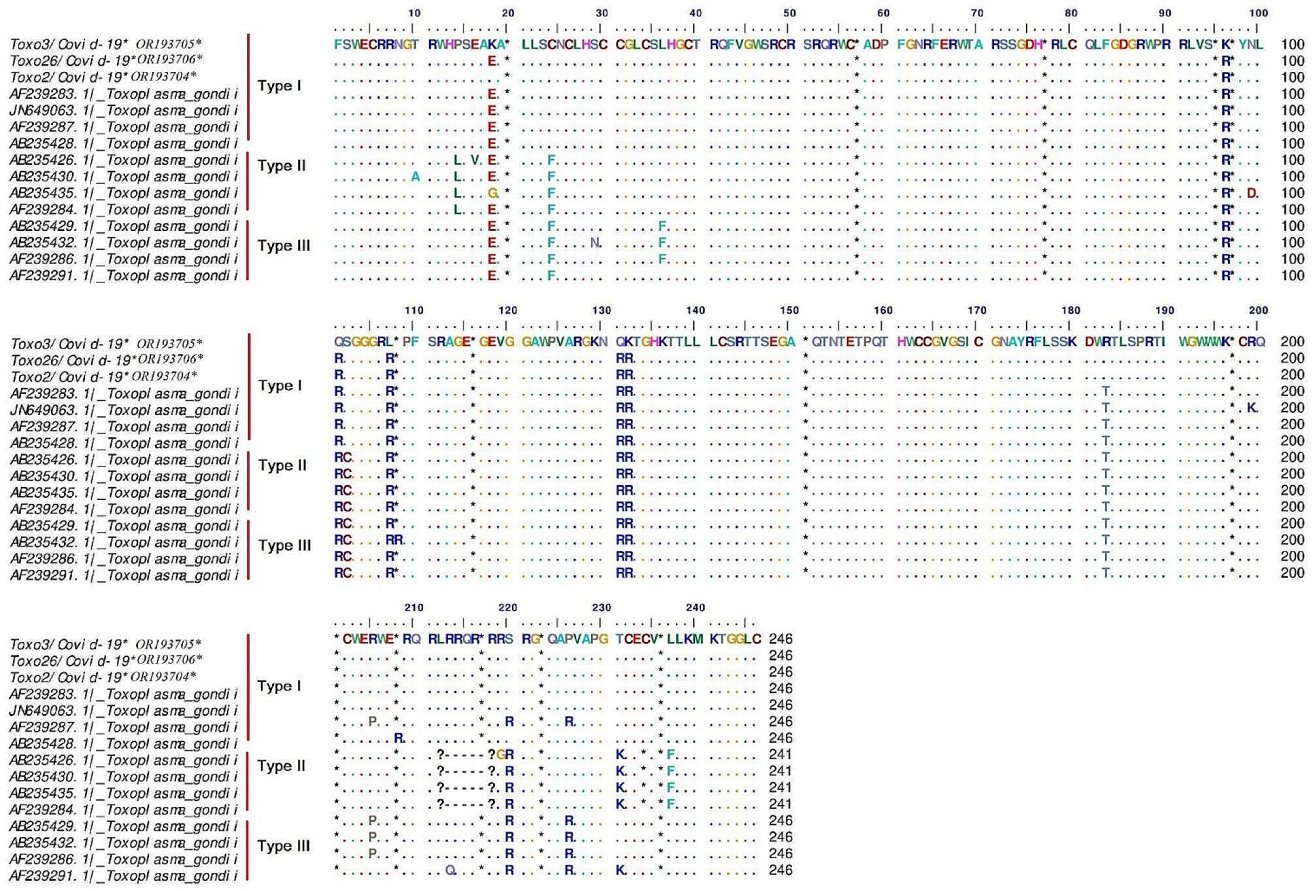
Multiple alignments of *T. gondii* GRA6 sequences. The non-synonymous (amino acid) substitutions of *T. gondii* sequences (Toxo2/3/26-COVID-19*) including five codon positions; 19, 96, 101, 107 and 132 were observed in the COVID-19 patients

The phylogenetic tree revealed the type I genotype of *Toxoplasma* (Toxo2/3/26-COVID-19; Accession nos; **OR193704**–**OR193706**) obtained from COVID-19 patients, placed in their specific clades (Fig. 3). The genomic analysis of the GRA6 sequences of *T. gondii* showed genetic diversity (Hd: 0.710) including three haplotypes; However, the Nd value was low (0.00631) (Table 2). Based on multiple alignment analyses of the GRA6 gene, five amino acid substitutions (non-synonymous) of *T. gondii* (codon positions; 19 (lysine (K) instead of glutamic acid (E)), 96 (K instead of arginine (R)), 101(R instead of glutamine (Q)), 107 (leucine (L) instead of R) and 132 (Q instead of R) were observed in the COVID-19 sequences (Toxo2/3/26-COVID-19*) (Fig. 4).

**Table 2:** Diversity indices of *T. gondii* obtained from COVID-19 patients in Tabriz, Northwest Iran.

**Area**	**City**	**N**	**Hn**	**Hd ± SD**	**Number of segregating sites**	**Nd (π)**
**Northwest Iran**	**Tabriz**	6	3	0.710 ± 0.272	7	0.00631

N: number of isolates; Hn: number of haplotype; Hd: haplotype (gene) diversity and Nd: nucleotide diversity

## Discussion

Bi-functional effects in the relationship between *T. gondii* and COVID-19 patients have been stated by several studies [18–21]. Current results showed a relatively high seroprevalence of anti-*Toxoplasma* IgG antibody (35.7%) among the COVID-19 patients in Tabriz; however, no meaningful association was observed between *Toxoplasma* seropositivity in COVID‑19 patients compared to healthy subjects.

A study reported that overall mortality in moderate or severe COVID-19 disease was associated with a positive anti-*Toxoplasma*’s IgG titer in Mazandaran, Northern Iran. However, no significant association was found between *T. gondii* infection and COVID-19 severity [17]. Geraili et al. (2022) have also shown that acute and latent toxoplasmosis infections circulate among COVID-19 patients in Golestan Province, Northern Iran. Furthermore, the prevalence of anti-*T. gondii* IgM and IgG antibodies were 5.0% and 26.1%, respectively, in COVID-19 patients. However, no significant associations have been found between *T. gondii* infection and COVID-19 severity [28].

On the other hand, Sharaf-El-Deen (2021) showed that toxoplasmosis through over-expression of lymphocytic PD-1 can be considered an independent risk factor for the severity of COVID-19 [15]. Roe (2021) pointed out that SARS-CoV-2 in synergy with active toxoplasmosis, intensifies the clinical symptoms in COVID-19 patients and leads to a challenging public health concern in all around the world [29].

Roe (2021) reported the association between *T. gondii* infection and higher mortality in COVID-19 patients with schizophrenia in Germany [30]. Nevertheless, another study did not confirm a strong association between *T. gondii* infection and susceptibility to COVID‑19 [21]. In a recent study in the Mexican population, the prevalence of IgG and IgM anti*-Toxoplasma* antibodies was demonstrated in 27.34% and 13.6% of COVID-19 patients, respectively [31]. A study conducted on the Czech and Slovak populations showed that toxoplasmosis is not considered a risk factor for COVID-19 and *Toxoplasma*-infected patients [16].

The current results showed that risk factors such as close contact with stray cats and consumption of raw vegetables can increase the transmission of *T. gondii* infection to COVID-19 patients. Hence, educational policies such as thoroughly washing contaminated vegetables to remove oocysts and feeding cooked food to stray cats should be applied to COVID-19 patients with toxoplasmosis.

In this study, despite the low number of CD4^+^T cells, no severe clinical symptoms of active toxoplasmosis (lymphadenopathy, chorioretinitis and cerebral/pulmonary manifestations) were detected in COVID-19 patients infected with high titer of *T. gondii* IgG. However, Erol et al. (2022) reported secondary toxoplasmosis chorioretinitis with retinal detachment that developed shortly after COVID-19 infection [23].

*T. gondii* can cause latent toxoplasmosis in the brain, central nervous system (CNS) and muscles [32]. TH1-associated pro-inflammatory cytokines (cell-mediated immunity) are the main immunity responses against *T. gondii* infection [33]. According to this fact, a chronic inflammatory response against toxoplasmosis could exacerbate the severity of COVID-19.

Recent evidence shows that CD4 T cell and CD8 T cell depletion occurs during co-infection of coronavirus disease and toxoplasmosis, called “polyspecific T cell exhaustion” leading to overproduction of TH2 cytokines [34]. Subsequently, switching the TH1 response to TH2 could reactivate latent toxoplasmosis in COVID-19 patients.

CD4 T cell depletion increases CD8 T cell depletion because the lack of interleukin-21 signals secreted by CD4 T cells directly increases CD8 T cell depletion [35]. Consequently, loss of CD4 T cell functions due to CD4 T cell depletion leads to a reduction in interferon-γ levels, a crucial cytokine required to control both chronic and acute toxoplasmosis [34]. On the other hand, Lucas et al. (2020) demonstrated that helminths can alter the severity of COVID-19. Indeed, during helminth infections, host immune responses shift toward TH2 polarization with a controlled inflammatory component that reduces mortality/morbidity in COVID-19 patients [36].

In this study, high genetic diversity of *T. gondii* GRA6 sequences (including five codon substitutions) was found in COVID-19 patients. The emergence of haplotype diversity in latent toxoplasmosis should be considered in view of the emergence of treatment-resistant alleles and/or the development of pathogenesis, particularly in symptomatic COVID-19 patients with high disease severity.

In this study, we were unable to determine the level of *T. gondii* genetic diversity in COVID-19 patients infected with high titer of *T. gondii* IgM antibody. Furthermore, we could not authentically compare the haplotype substitutions of the GRA6 sequences of *T. gondii* between latent and active toxoplasmosis. One of the limitations of the present study was that the number of *T. gondii* sequences obtained from COVID-19 patients was small to infer large-scale genetic diversity.

The present study revealed that *T. gondii* type I infections are unequivocally circulating among COVID-19 patients in Tabriz, Northwest Iran. This study found an insignificant correlation between *Toxoplasma* infection and COVID-19 patients. The detection of *T. gondii* in COVID-19 patients will help to develop an epidemiological understanding of toxoplasmosis and implement preventive programs in the region. To make more accurate health decisions, multicenter investigations with a larger sample size of different ethnic groups of the Iranian population are needed.

### Electronic supplementary material

Below is the link to the electronic supplementary material.

Supplementary Material 1: Nested-PCR assay by targeting *T. gondii* B1

Supplementary Material 2: Nested-PCR assay by targeting *T. gondii* GRA6

## Data Availability

The datasets generated and/or analyzed during the current study are available in the NCBI database, [Accession numbers: **OR193704**–**OR193706**].
