# Supplementary material for: Genetic diversity and seroprevalence of Toxoplasma gondii in COVID‑19 patients; a first case-control study in Iran
Source: BMC Infect Dis. 2024 Jan 3;24:42. doi: 10.1186/s12879-023-08964-9 (PMC10763165; doi:10.1186/s12879-023-08964-9)
Supplement: Supplementary file 2 — Supplementary Material 2: Nested-PCR assay by targeting T. gondii GRA6 [file 12879_2023_8964_MOESM2_ESM.docx]

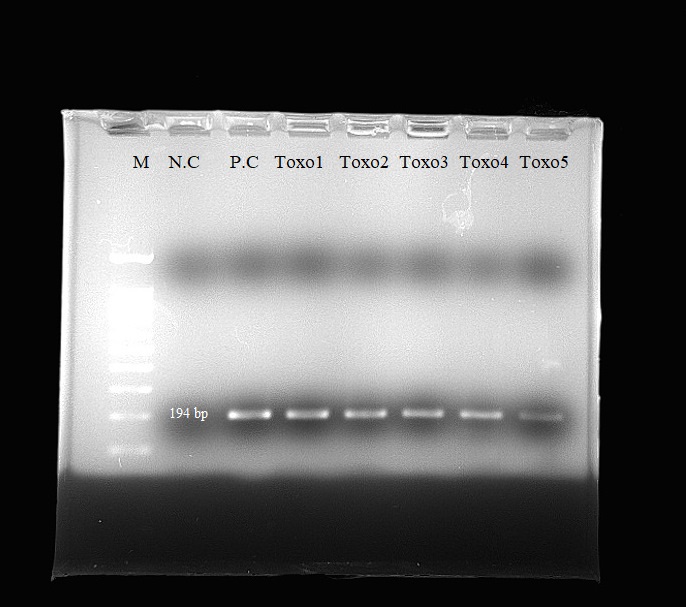


Supplementary Figure 2: Nested-PCR assay targeting *T. gondii* B1 (B: Toxo1-Toxo5: 194 bp). M; Ladder marker (size marker: 100bp), P.C; positive control, N.C; negative control.
